# Repeatability of Foveal Measurements Using Spectralis Optical Coherence Tomography Segmentation Software

**DOI:** 10.1371/journal.pone.0129005

**Published:** 2015-06-15

**Authors:** Irene Ctori, Byki Huntjens

**Affiliations:** Applied Vision Research Centre, The Henry Wellcome Laboratories for Vision Sciences, City University London, Northampton Square, London, EC1V 0HB, United Kingdom; Justus-Liebig-University Giessen, GERMANY

## Abstract

**Purpose:**

To investigate repeatability and reproducibility of thickness of eight individual retinal layers at axial and lateral foveal locations, as well as foveal width, measured from Spectralis spectral domain optical coherence tomography (SD-OCT) scans using newly available retinal layer segmentation software.

**Methods:**

High-resolution SD-OCT scans were acquired for 40 eyes of 40 young healthy volunteers. Two scans were obtained in a single visit for each participant. Using new Spectralis segmentation software, two investigators independently obtained thickness of each of eight individual retinal layers at 0°, 2° and 5° eccentricities nasal and temporal to foveal centre, as well as foveal width measurements. Bland-Altman Coefficient of Repeatability (CoR) was calculated for inter-investigator and inter-scan agreement of all retinal measurements. Spearman's ρ indicated correlation of manually located central retinal thickness (RT_0_) with automated minimum foveal thickness (MFT) measurements. In addition, we investigated nasal-temporal symmetry of individual retinal layer thickness within the foveal pit.

**Results:**

Inter-scan CoR values ranged from 3.1μm for axial retinal nerve fibre layer thickness to 15.0μm for the ganglion cell layer at 5° eccentricity. Mean foveal width was 2550μm ± 322μm with a CoR of 13μm for inter-investigator and 40μm for inter-scan agreement. Correlation of RT_0_ and MFT was very good (ρ = 0.97, P < 0.0005). There were no significant differences in thickness of any individual retinal layers at 2° nasal compared to temporal to fovea (P > 0.05); however this symmetry could not be found at 5° eccentricity.

**Conclusions:**

We demonstrate excellent repeatability and reproducibility of each of eight individual retinal layer thickness measurements within the fovea as well as foveal width using Spectralis SD-OCT segmentation software in a young, healthy cohort. Thickness of all individual retinal layers were symmetrical at 2°, but not at 5° eccentricity away from the fovea.

## Introduction

The arrival of Optical Coherence Tomography (OCT) has changed the way that retinal pathology is diagnosed and managed. OCT imaging allows non-invasive cross-sectional imaging of the human retina [1]. Good correlation with retinal histology [2–4] pertains OCT technology to the clinical diagnosis of a variety of ocular pathologies [5–8] based on quantitative evaluation of retinal thickness measurements in-vivo [9–11]. Newer spectral domain (SD-OCT) methods offer faster acquisition time and improved image resolution compared to older time-domain OCT techniques [12,13]. In addition, automated retinal thickness measurement techniques are a time-efficient way to investigate retinal thickness change over time [14]. Repeatability and reproducibility of automated total retinal thickness measurements using SD-OCT has been demonstrated in healthy individuals [15,16] as well as those with ocular pathology [17–22]. This has enabled the definition of levels at which true clinical change can be distinguished from measurement variability. However, OCT instruments employ a variety of segmentation algorithms within their software platforms so that measurements cannot be directly compared between instruments [23,24]. It is therefore important to establish the repeatability and reproducibility of retinal measurements for each OCT device being used for clinical diagnosis and treatment protocol designs [9–11].

According to the configuration of the Spectralis SD-OCT (Heidelberg Engineering, Heidelberg, Germany), one pixel represents 3.9μm axially and 6μm laterally [25]. It features Automatic Real Time (ART), a setting that improves image quality by averaging multiple B-scans to reduce noise and Tru-Track, an eye-tracking device that improves scan reproducibility [26]. Compared to other OCT instruments, the Spectralis SD-OCT presents the highest reproducibility of automated crude central foveal thickness measurement [27,22]. Very recently, Heidelberg Engineering launched an update to the Spectralis SD-OCT Heidelberg Eye Explorer mapping software (version 6.0c) that allows automatic segmentation of individual retinal layers.

This study reports inter-investigator and inter-scan repeatability of thickness of eight individual retinal layers including the inner and outer plexiform and nuclear layers along with combined inner retinal layer thickness and overall retinal thickness at manually derived axial and lateral foveal locations. Repeatability of foveal width measurements is also investigated. All measurements are derived from Spectralis SD-OCT scans using the newly available Spectralis retinal layer segmentation software.

## Methods

### Study protocol

The study included 40 healthy volunteers and took place at the Division of Optometry and Visual Science, City University London from October to December 2013. The inclusion criterion was logMAR visual acuity better than 0.3 log units in the eye being tested. Exclusion criteria were ocular pathology including corneal disease, macular disease and fundus myopicus, medication that may affect retinal function and previous eye surgery, including refractive laser correction. For each volunteer, the eye with the best logMAR acuity was selected as the test eye. Mean spherical error (MSE), calculated as sphere plus half of the cylinder[28] (average of five autorefractor readings), and mean keratometry measurements (average of three horizontal and vertical readings) were obtained using the Topcon TRK-1P autorefractor (Topcon, Tokyo, Japan). Two experienced investigators (A and B) each derived foveal measurements from Spectralis SD-OCT scans, using the techniques described below. Investigators A and B both obtained measurements from the first scan of each participant (1A and 1B respectively), and investigator B took measurements from the second scan (2B). For repeat measurements, each investigator was masked to their initial or the other investigator’s results. Tomograms were measured in a random order to minimize this potential source of bias.

### SD-OCT scan acquisition

All scans were obtained without pupil dilation [29–31] in a dark room using the Spectralis SD-OCT device. As recommended by manufacturer instructions, each participant’s mean keratometry value was inserted into the Spectralis software prior to scan acquisition [32]. Two consecutive 20° x 5° volume scans (49 B-scans 30 microns apart, ART 16 frames including 1024 A scans) were taken for the test eye within a single visit, without setting the first scan as a reference. The participant was instructed to sit back from the device between scans. Each time, the investigator focused the infrared fundus image according to the participant’s MSE. Central fixation was monitored via the live fundus image and scan quality was accepted above 25 decibels (dB), in accordance with the manufacturer guidelines.

### Foveal measurements

Foveal measurements from each SD-OCT scan were performed using the inbuilt Spectralis mapping software, Heidelberg Eye Explorer (version 6.0c). The new Spectralis segmentation software was used to obtain individual retinal layer thickness measurements including: overall retinal thickness (RT), retinal nerve fibre layer (RNFL), ganglion cell layer (GCL), inner plexiform layer (IPL), inner nuclear layer (INL), outer plexiform layer (OPL), outer nuclear layer (ONL), retinal pigment epithelium (RPE), inner retinal layer (IRL) and photoreceptor layer (PR). Measures of foveal width were also evaluated, as well as the correlation of manual and automated measures of central retinal thickness. In addition, we explored the horizontal symmetry from the foveal centre of the thickness of the individual retinal layers.

**Fig 1:**
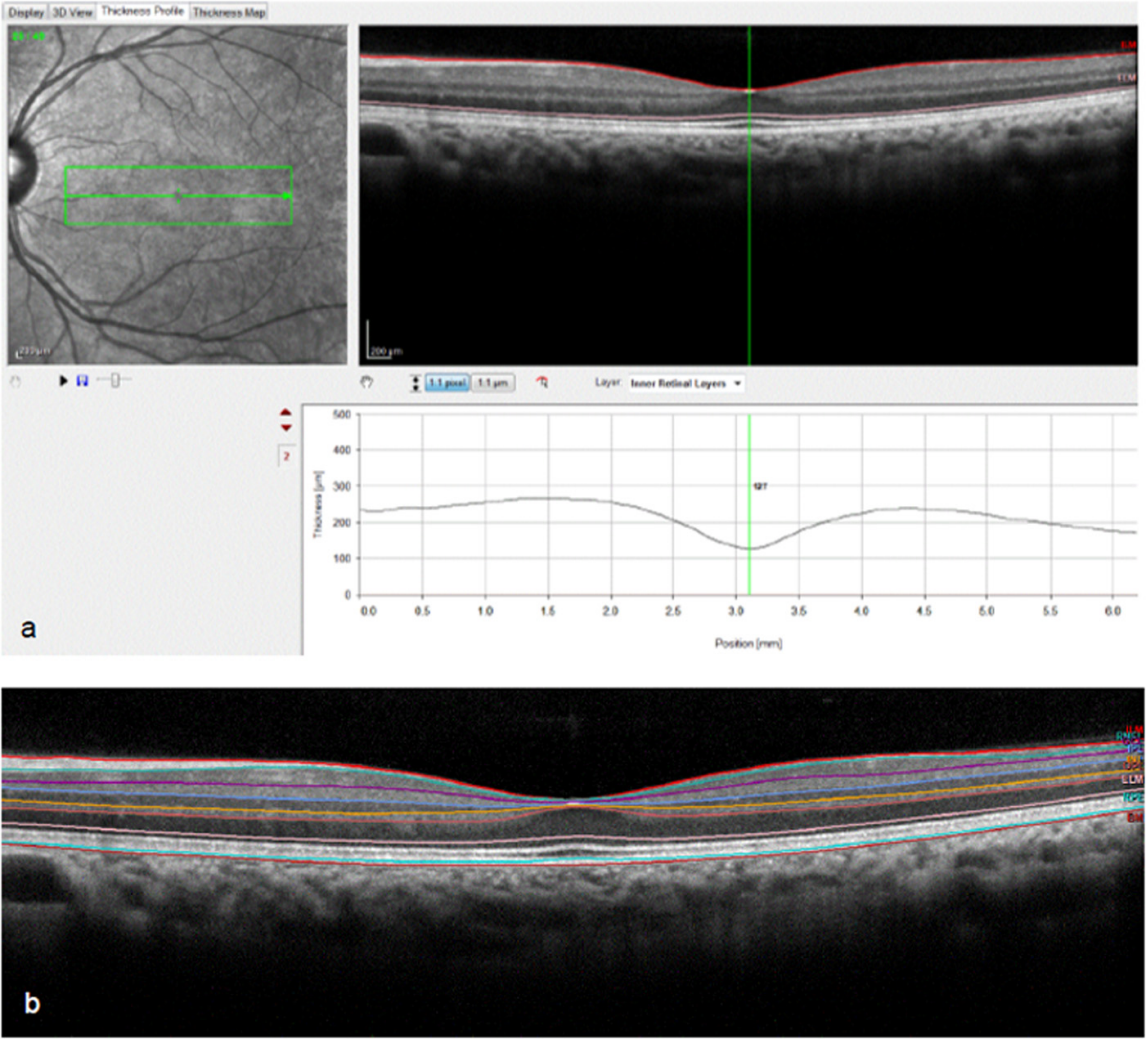
1a and b. Central retinal thickness and layer segmentation by Spectralis SD-OCT software. The Spectralis software displays overall retinal thickness as the vertical distance between the vitreoretinal interface and Bruch’s membrane. Using the thickness profile, the foveal reflex was bisected by the software caliper, and the thickness of the individual layers was recorded in microns (a). Segmentation of the individual retinal layers can be seen in the lower image (b).

No manual adjustments to B-scan retinal layer segmentation were made prior to measurements being taken. For each scan, the foveal centre was identified as the frame including the brightest foveal reflex [33,34]. As suggested by Mohammad *et al*., when a bright reflex was absent or present in two or more frames, the frame containing the thickest outer segment layer was chosen [35]. At the point where the software caliper bisected the foveal reflex, individual layer thickness (RT, RNFL, GCL, IPL, INL, OPL, ONL, RPE, IRL and PR) was recorded in microns (Fig 1A). The software displays overall retinal thickness as the vertical distance between the vitreoretinal interface and Bruch’s membrane (Fig 1B).

**Fig 2:**
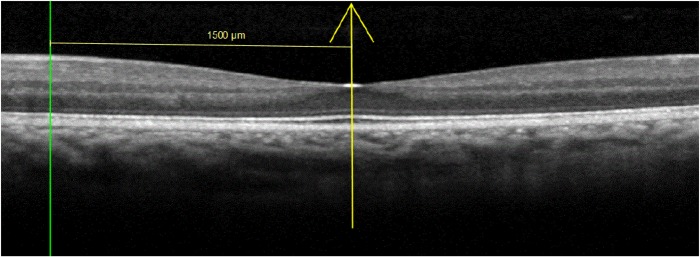
Positioning of software caliper for lateral retinal thickness measurement.

Thickness of each retinal layer was also measured at 2° and 5° eccentricity away from the fovea. In order to locate these lateral positions on the tomogram, the eccentricities in degrees were converted into microns based on each individual’s OCT scan length. For example, given that the scan length (in millimeters, mm) generated by the Spectralis represents 20°, the lateral equivalent in microns of 2° would be 2*(scan length/20). The inbuilt software caliper was set at the appropriate lateral distance perpendicular to the vertical caliper bisecting the foveal reflex and thickness of each retinal layer recorded from the retinal thickness profile (Fig 2). Lateral measurements were taken nasal to the fovea for all tomograms. In addition, temporal retinal thickness measurements were also obtained for the first scan of each participant to assess horizontal symmetry.

**Fig 3:**
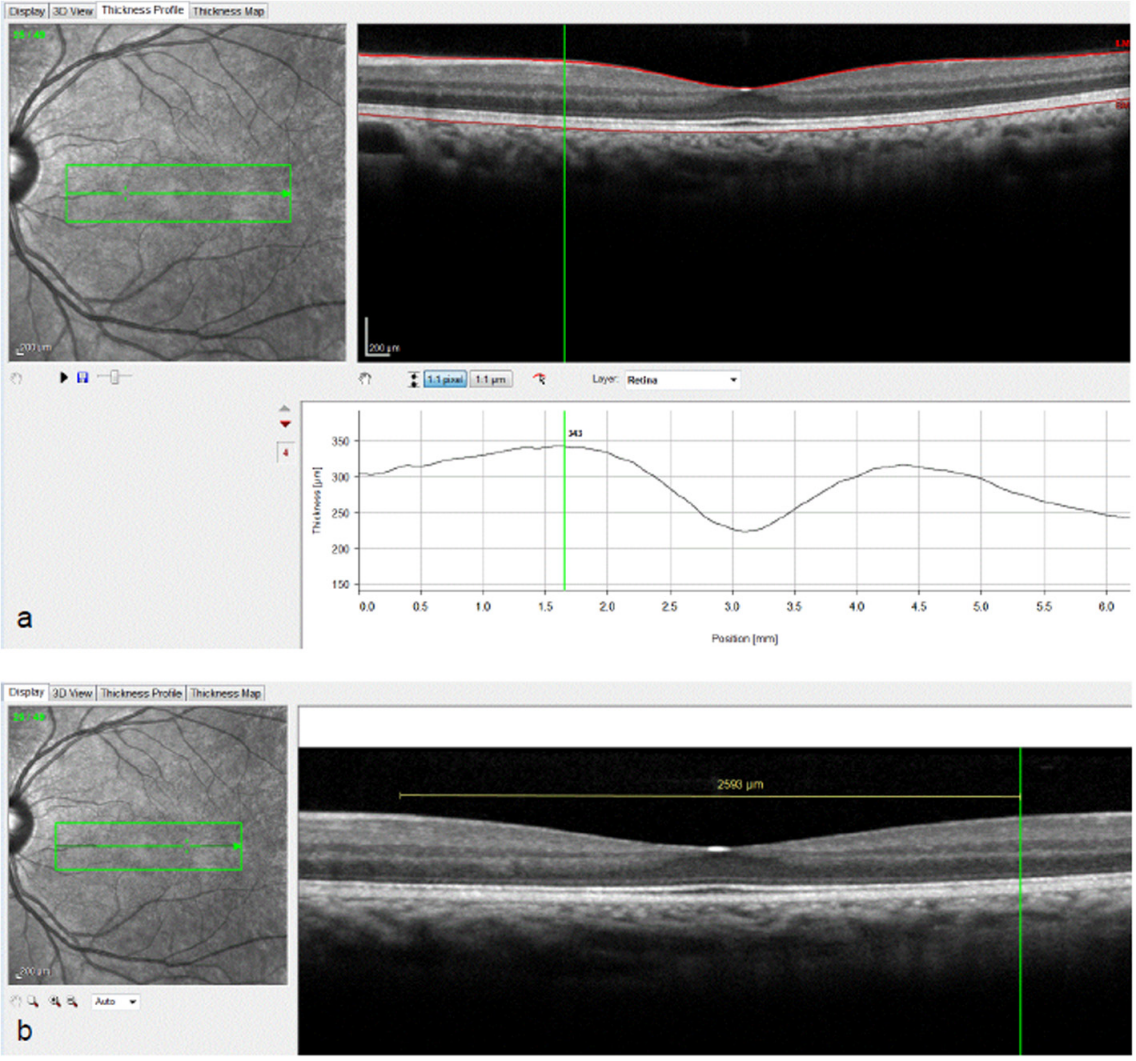
a and b. Measurement of foveal width. Maximum retinal thickness nearest to the foveal reflex on nasal (a) and temporal side identified from the thickness profile. Foveal width was measured in microns using the inbuilt manual calipers (b).

Using the inbuilt manual calipers, foveal width was measured in microns as the horizontal distance between foveal crests [11,30,33,36], identified as the maximum retinal thickness nearest to the foveal reflex on the nasal and temporal side (Fig 3A and 3B).

The Spectralis mapping software also generates automated measures of retinal thickness based on analyses of the central and inner 1000, 3000 and 6000μm subfields as defined by the Early Treatment Diabetic Retinopathy Study [37]. From this, the central minimum retinal thickness value was recorded as the minimum foveal thickness (MFT) for each scan. Central foveal thickness of each retinal layer (CFT), corresponding to the average thickness of all points within the central ETDRS zone of 1000μm diameter, was also recorded.

### Ethical approval and consent

Approval for the study was obtained from the Optometry Research & Ethics Committee City University London. All subjects gave written informed consent conforming to the tenets of the Declaration of Helsinki.

### Statistical analysis

All statistical analyses were performed using SPSS version 22.0 for Windows (SPSS Inc., Chicago, USA). Values in the text and tables are presented as the mean ± standard deviation (SD). Preliminary analyses were performed to ensure no violation of the assumptions of normality, linearity and homoscedasticity. The CoR was calculated as *1*.*96s*, where *s* is the SD of the difference between pairs of measurements [38]. Limits of agreement (LoA) were calculated as the mean difference between two sets of data ± CoR. The LoA indicate the range within which 95% of the differences between measurements will lie [38–40].

We calculated the inter-investigator agreement of the thickness of each retinal layer and also foveal width measurements from the first scan (1A versus 1B). The inter-scan CoR for the same retinal measurements taken by investigator B was also calculated (1B versus 2B). We determined the correlation of manual location of central retinal thickness (RT_0_) and MFT using Spearman's Rank Correlation coefficient, ρ. The independent t-test was used to assess difference between nasal and temporal retinal layer thickness. Statistical significance was accepted at P < 0.05.

## Results

The study group included 40 participants (12 males and 28 females) with a mean age of 21.1 ± 3.1 years (range 18 to 36 years). Mean MSE was -1.70 ± 2.32DS (ranging from -10.00DS to +0.50DS) and mean keratometry was 7.83 ± 0.30mm (ranging from 7.16 to 9.05mm). There was no significant difference in mean image quality between scan 1 (38 ± 4dB) and scan 2 (38 ± 3dB; P = 1.00).

**Fig 4:**
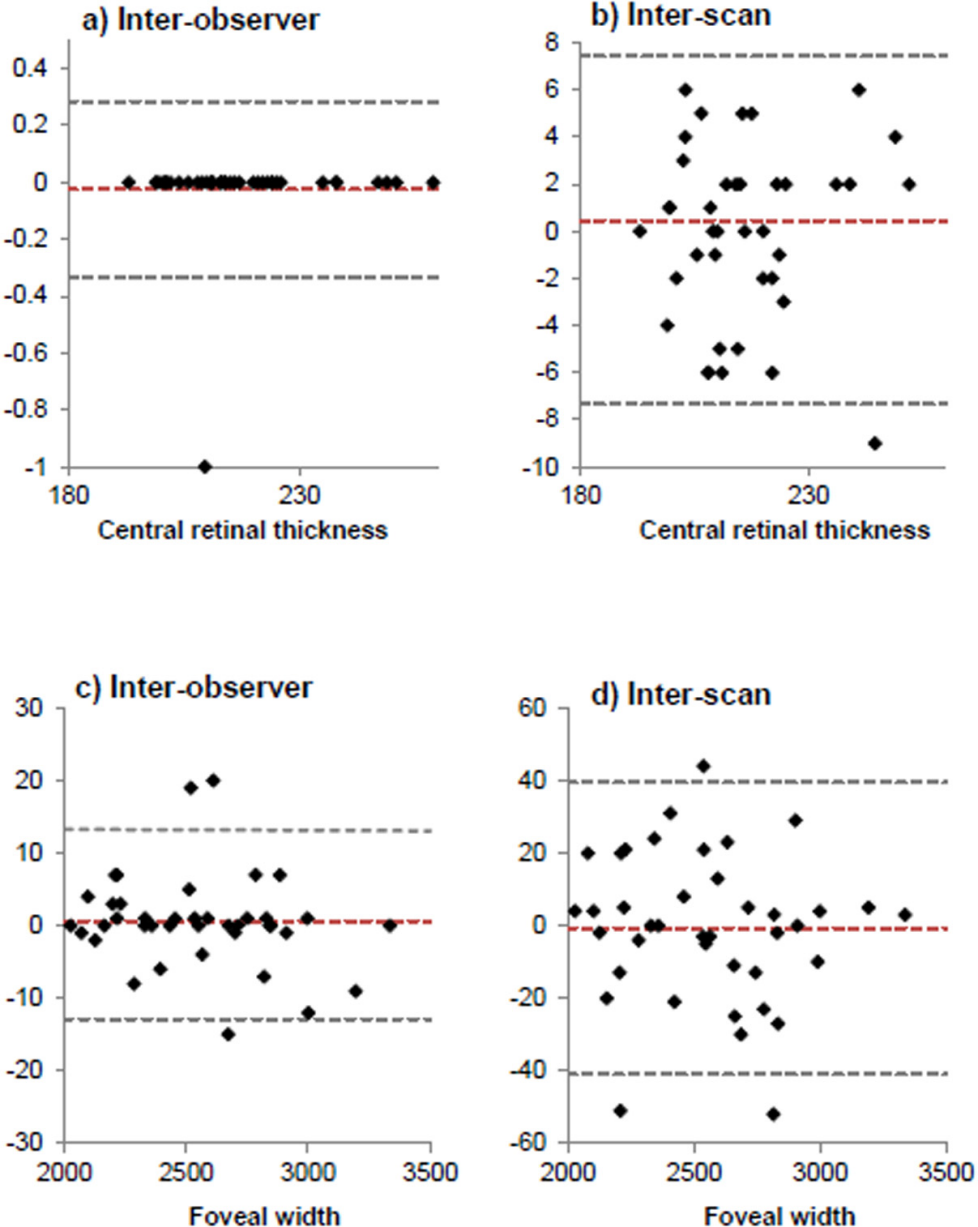
a-d. Bland-Altman plots to show a) Inter-observer agreement of central retinal thickness; b) Inter-scan agreement of central retinal thickness; c) Inter-observer agreement of foveal width; d) Inter-scan agreement of foveal width. All measurements presented in microns. Red line indicates mean difference, d between values. Limits of Agreement (d+1.96s) represented by upper and lower grey dashed lines respectively.

Repeatability of thickness of individual retinal layer measurements are presented in Table 1 (inter-investigator) and Table 2 (inter-scan), with the mean difference and CoR values for each layer at 0°, 2° and 5° nasal eccentricity as well as the CFT given. Mean overall retinal thickness was 217 ± 16μm at 0°, 296 ± 27μm at 2° and 350 ± 16μm at 5° nasal to foveal centre, with respective CoR values of 0.3, 3.2 and 0.5μm for inter-observer and 7.4, 8.5 and 7.6μm for inter-scan agreement. Mean foveal width was 2550μm ± 322μm with mean difference of 0.60μm and CoR of 13μm for inter-investigator and mean difference of -0.70μm and CoR of 40μm for inter-scan agreement. Bland-Altman plots are presented in Fig 4.

**Table 1:** Inter-observer agreement of thickness of retinal layers in microns.

I)	Eccentricity from foveal centre (degrees)					
II)	0		2		5	
Retinal layer	Mean difference	CoR	Mean difference	CoR	Mean difference	CoR
III) Retina	-0.025	0.31	-0.425	3.20	-0.075	0.52
IV) Retinal nerve fibre layer	-0.025	0.31	0.225	3.91	-0.10	0.74
V) Ganglion cell layer	-0.05	0.43	-0.35	2.41	-0.025	0.31
VI) Inner plexiform layer	0.025	0.31	-0.10	1.07	-0.025	0.31
VII) Inner nuclear layer	0.125	1.27	-0.15	1.14	0.00	0.44
VIII) Outer plexiform layer	0.025	0.54	0.075	1.03	-0.025	0.54
IX) Outer nuclear layer	-0.125	1.73	-0.075	2.36	0.00	0.44
X) Inner retinal layer	-0.025	0.70	-0.475	3.20	-0.075	0.93
Photoreceptor layer	-0.05	0.43	-0.025	1.13	0.075	0.93
XI) Retinal pigment epithelium	0.00	0.77	0.05	1.08	0.025	0.31

Retinal thickness refers to thickness from the inner limiting membrane to the external limiting membrane. Limits of Agreement are equal to the mean difference ± Coefficient of Repeatability (CoR).

**Table 2:** Inter-scan agreement of thickness of retinal layers in microns at 0, 2 and 5° from foveal centre.

XII)	Eccentricity from foveal centre (degrees)							
XIII)	0		2		5		CFT	
Retinal layer	Mean difference	CoR	Mean difference	CoR	Mean difference	CoR	Mean difference	CoR
XIV) Retina	-0.35	7.4	-0.423	8.46	0.5	7.57	-0.08	3.7
XV) Retinal nerve fibre layer	0.18	3.1	0.75	8.42	-0.85	10.0	-0.05	1.6
XVI) Ganglion cell layer	-0.43	4.4	-1.00	7.13	-0.83	15.0	-0.18	1.8
XVII) Inner plexiform layer	-0.53	5.7	0.03	7.29	-0.20	9.2	-0.32	3.6
XVIII) Inner nuclear layer	-0.23	5.0	0.75	9.74	0.35	14.1	-0.03	2.0
XIX) Outer plexiform layer	-0.90	8.9	-0.25	10.7	0.80	14.8	-0.2	6.0
XX) Outer nuclear layer	1.85	14.7	0.63	13.9	-0.28	4.92	-0/05	6.9
XXI) Inner retinal layer	0.18	12.0	0.63	14.1	-0.03	7.97	-0.20	7.7
Photoreceptor layer	-0.13	13.2	0.53	12.5	1.05	7.36	0.53	4.9
XXII) Retinal pigment epithelium	0.15	11.6	0.08	8.54	0.45	4.57	0.18	2.1

Retinal thickness refers to thickness from the inner limiting membrane to the external limiting membrane. Limits of Agreement are equal to the mean difference ± Coefficient of Repeatability (CoR).

The automated measure of MFT showed a mean of 216 ± 15μm for the first scan and 217 ± 15μm for the repeated scan. MFT mean difference between scans was 0.33μm, with CoR of 2.19 and LoA from -1.87 to 2.52μm. There was excellent correlation between automated MFT and the manual RT_0_ measurements taken from investigator B's analysis of the first scan (ρ = 0.97, P < 0.0005).

The mean thickness of the individual retinal layers at the foveal centre and at 2° and 5° eccentricity are given in Table 3. While there was no significant difference in thickness of all individual retinal layers at 2° nasal compared to temporal to fovea (P > 0.05) this was not true at 5° eccentricity, whereby the thickness of RT, RNFL, GCL, INL, ONL and IRL were significantly increased nasally compared to temporally (Table 3).

**Table 3:** Mean thickness of individual retinal layers at foveal centre and at 2 and 5 degrees eccentricity nasal and temporal to fovea.

		Eccentricity from foveal centre (degrees)					
		2			5		
Retinal layer		Mean+SD		P-value	Mean+SD		P-value
Retina	nasal	296	27	0.80	350	16	**<0.0005**
	temporal	298	19		321	14	
XXIII) Retinal nerve fibre layer	nasal	17	4	0.10	22	5	**<0.0005**
	temporal	19	5		13	4	
Ganglion cell layer	nasal	26	9	0.99	60	5	**<0.0005**
	temporal	26	6		50	8	
Inner plexiform layer	nasal	29	7	0.23	47	5	0.15
	temporal	31	6		45	5	
Inner nuclear layer	nasal	25	7	0.06	42	5	**<0.0005**
	temporal	28	6		38	7	
Outer plexiform layer	nasal	28	7	0.97	29	5	0.84
	temporal	28	5		29	6	
Outer nuclear layer	nasal	80	12	0.43	72	9	**<0.0005**
	temporal	82	12		67	8	
XXIV) Inner retinal layer	nasal	208	27	0.43	271	15	**<0.0005**
	temporal	212	19		241	14	
Photoreceptor layer	nasal	88	8	0.09	80	3	0.06
	temporal	85	6		79	3	
Retinal pigment epithelium	nasal	17	3	0.09	13	2	0.30
	temporal	16	3		13	2	

P-value of independent t-test between nasal and temporal shown.

## Discussion

We investigated Spectralis SD-OCT repeatability and reproducibility of manually derived and automated axial, as well as lateral foveal measurements in young healthy individuals. To our knowledge, this is the first report of repeatability and reproducibility of thickness measurements of each of eight individual retinal layers at the centre of the fovea as well as at two lateral positions derived using the newly available Spectralis segmentation software. Manual measurements of RT_0_ (217 ± 16μm) and automated MFT (216 ± 15μm) in the current study compare well with those obtained in a study using the Spectralis OCT device in which a mean automated foveal thickness of 228 ± 11μm of forty subjects aged 19 to 50 years was reported [23]. Our results show that inter-observer CoR values were less than 4μm for all individual layer thicknesses. The CoR values at 2° were greater than at 0° or 5° eccentricity with the greatest difference in the RT, RNFL, GCL and IRL, most likely due to software algorithm errors. Compared to inter-observer agreement, inter-scan CoR values were greater and varied across individual layers, up to a maximum of 15μm for the GCL at 5° eccentricity nasal to the foveal centre. LoA for RT_0_ were narrower for inter-observer compared to inter-scan measurements (Fig 4). There was one outlier in each case that could not be explained. In agreement with an earlier report [19], there did not appear to be any relationship between mean central retinal thickness or foveal width and repeatability. It has been shown previously that retinal thickness measurements may be affected by OCT image quality below the acceptable range stated by the OCT manufacturer [41]. This should be taken into account when examining individuals in whom the image quality is worse, for example due to cataract. Mean image quality of all scans in the current study was excellent at 38dB eliminating this source of error. We did not use the reference setting option to acquire the second scan. An earlier study showed that this may unlikely affect the reproducibility of RNFL thickness in normal eyes [42]; however, this should be confirmed for all retinal layers.

A strength of our study is that all measurements were obtained from scans that had individual ocular biometry taken into account. Individual scan lengths are generated by the Spectralis software based on the subject’s corneal curvature and refractive error as well as a non-modifiable pre-set axial length to minimise the effects of lateral magnification caused by the optics of the eye [32]. While we did not perform a subjective refraction on each participant, it has been shown that using an autorefractor to approximate refractive error is an accepted method [43]. In addition, optical defocus of two diopters has minimal effect on retinal thickness measurements obtained with the Spectralis [41].

It has been shown that the centre of the fovea assumed by OCT instruments and the retinal locus of fixation do not always correspond [44,45], with deviations of approximately 60 ± 50μm between fixation and the centre of the foveal avascular zone [46]. In order to correlate some measure of visual function at fixation (e.g. visual acuity or macular pigment) with retinal anatomy at the corresponding retinal locus, it may be more appropriate to manually locate the fixation point for foveal thickness measurements. Indeed, visual inspection of OCT images with manual identification of the foveal centre was the preferred method in a study quantifying foveal thickness and visual acuity in albinism [35]. However, the repeatability of manually derived lateral and axial retinal measurements is less well documented: one study was based on manual measurements of a model eye [24], while another study explored the repeatability of manual sub-foveal choroidal thickness measurements [10]. We have shown excellent correlation between automated MFT and manually located RT_0_ measurements (ρ = 0.97, P < 0.0005). The low CoR values for RT_0_ (<1 μm inter-observer and <8μm inter-scan) show that the method of manually selecting the position at which to measure central retinal thickness is robust to inter-investigator and inter-scan variability. Additionally, in the current study, both investigators independently selected the same tomogram for analysis using the protocol described in the methods in all cases.

Repeatability of automated MFT and CFT has shown to vary across OCT devices and also depend on the scan protocol employed [47]. We have shown high reproducibility of automated macular thickness measurements (MFT) using the Spectralis to obtain high resolution 20° x 5° volume scans (49 B-scans 30 microns apart, ART 16 frames, 1024 A scans), indicated by the inter-scan CoR of 2.19μm. This is in accordance with a previous report in which the LoA were -2.49 to 3.77μm for inter-observer agreement of mean macular thickness measures using the Spectralis [27]. The inter-scan CoR of 3.7μm for CFT also compares well with a study in which a CoR value of 2.69μm for mean macular thickness across the central 1000μm diameter was reported using the Stratus OCT device [31]. However, in an investigation involving 50 subjects with diabetic macula oedema, a higher CoR of 8.03μm was reported for Spectralis SD-OCT automated central subfield retinal thickness measurements [18]. This suggests that ocular pathology increases the level at which true clinical change has occurred as opposed to measurement variability most likely due to fixation problems. In addition, the CoR for retinal thickness in subfields surrounding the foveal centre ranged from 3.97 to 7.23μm [18]. Caution must therefore be taken when considering the level at which clinical change is deemed to occur in individuals with retinal pathology and low vision [18], and for retinal thickness changes occurring away from the centre of the fovea [48].

To our knowledge there are no reports of repeatability of manually derived lateral SD-OCT scan measurements in human subjects. We found a considerably large mean foveal width of 2550μm ± 322μm. Foveal pit diameters up to 2510μm have been reported using the Cirrus OCT [49] based on measuring the foveal pit from rim-to-rim using an automated MatLab algorithm [50]. Comparing foveal width between studies is challenging due to its variable definition. The mean foveal diameter of sixty healthy subjects was found 1244 ± 211μm measured between the points at which the nerve fibre layer ends, and 1371 ± 215μm when measured in the same subjects from foveal crest-to-crest [30]. Nevertheless we found a mean difference in foveal width of just 0.60μm between measurements obtained independently by the two investigators. This is much smaller than the difference of -14μm found in a study using the Cirrus OCT [49]. Estimation of the reproducibility of lateral foveal width measurements obtained from two scans of the same participant acquired within one visit by investigator B yielded a CoR of 40μm. This relatively large inter-scan CoR should be taken into account when investigating differences in foveal diameter between individuals, or longitudinally with time. Of note, LoA were wider for inter-scan compared to inter-observer measures of foveal width. The three outliers in both cases could not be explained. Nonetheless, when investigating change over time in a clinical setting a baseline scan image is usually set as a reference and repeated scans are subsequently compared to this. It is expected that this would improve the CoR for the lateral measurements [51].

Few studies have quantitatively assessed both inner and outer retinal morphology of the foveal pit. An earlier study reported circular symmetry of the outer retina (from the external limiting membrane to Bruch's membrane) at low eccentricities [52]. Our results indicate that the individual inner and outer retinal layers are all symmetrical at low eccentricities. In contrast, at 5° eccentricity there were significant differences in thickness of RT, RNFL, GCL, INL, ONL and IRL (Table 3). Asymmetry of the RNFL and GCL is not surprising given the distribution of the RNFL, with the thinnest peripapillary RNFL thickness found within the papillomacular bundle [42,53]. The evaluation of inner and outer retinal layer symmetry in the current study may be useful in future investigations of foveal morphology [54]. Choroidal thickness [10] and the length of the photoreceptor layers [35] are increasingly being used as both diagnostic and visual prognostic indicators in a variety of retinal disease states such as albinism [35]; and neuronal GCL loss has been evaluated in eyes of patients with multiple sclerosis [55]. Further work is needed however to estimate the reliability of measurements in eyes with macular pathology where poor fixation and disruptions in retinal morphology might make these measurements more variable [56].

We estimated the measurement error of our manually derived axial and lateral retinal measurement methods. Measurement error may be caused by instrument and software algorithm errors as well as operator error. Our results show that manually finding the location at which to extract central retinal thickness measurements is robust to inter-investigator repeatability. We also showed good reproducibility of individual retinal layer thickness measurements obtained from two scans acquired within a single visit. The inter-observer CoR values are actually smaller than the digital axial resolution of 3.9μm achievable with high resolution Spectralis SD-OCT (Spectralis technical guidelines) [25], indicating that there is very good repeatability of manual axial retinal thickness measurements between two observers looking at the same scan.

## Conclusion

Our findings show excellent repeatability and reproducibility of thickness measurements of each of eight individual retinal layers at manually derived axial and lateral foveal locations obtained using new Spectralis SD-OCT segmentation software in a young, healthy cohort. The inter-observer CoR values for each retinal layer give an indication of the level at which thickness and foveal width variation is indicative of true difference as opposed to measurement variability. The inter-scan CoR values signify the level at which change over time in axial and lateral measurements within an individual can be considered when the baseline reference scan feature of the Spectralis is not utilised. The method of manually selecting the position at which to measure central retinal thickness is robust to inter-investigator and inter-scan variability. We have demonstrated excellent correlation between automated and manually derived central retinal thickness measurements. Additionally, we have shown that the individual retinal layers are horizontally symmetrical at 2°, but not at 5° eccentricity. These results could provide valuable information for future studies involving foveal morphology specifically examining the individual retinal layers.
